# Awareness of resuscitation guidelines in pregnant women

**DOI:** 10.1007/s12471-017-0981-4

**Published:** 2017-03-29

**Authors:** S. Calle, M. De Leeuw, N. Mpotos, P. Calle, B. De Turck

**Affiliations:** 1 Faculty of Medicine and Health Sciences, Ghent University, De Pintelaan 185, 9000 Ghent, Belgium; 2 Forensic Pathology Department, Ghent University, De Pintelaan 185, 9000 Ghent, Belgium; 3 Department of Emergency Medicine, Sint Lukas General Hospital, Groenebriel 1, 9000 Ghent, Belgium; 4 Faculty of Medicine and Health Sciences, University of Antwerp, Universiteitsplein 1, 2610 Wilrijk, Belgium; 5 Department of Emergency Medicine, Maria Middelares General Hospital, Buitenring Sint-Denijs 30, 9000 Ghent, Belgium

We welcome this letter to the editor in reaction to our article ‘A fatal combination of situs inversus, pregnancy and cardiac arrest treated with an automated external defibrillator’.

The author correctly points out the importance of left lateral tilt and early perimortem delivery of the foetus via Caesarean section. As the length of a Rhythm Puzzle is limited, the scope of our article was restricted to the use of the automated external defibrillator.

We only want to add that the European Resuscitation Council elaborates on this issue in its Guidelines for Resuscitation 2015 (reference: Resuscitation 2015;95:184–186).

